# From “viewing” to “participation”: a study on the mechanisms underlying consumption behavior among sports streaming audiences

**DOI:** 10.3389/fpsyg.2026.1941880

**Published:** 2026-09-28

**Authors:** Ziqi Wang, Simin Chen, Feng Zhai

**Affiliations:** China University of Mining and Technology, Xuzhou, China

**Keywords:** consumption behavior, emotional responses, motivation for watching sports events, psychological belonging, sports spectatorship

## Abstract

**Background:**

Driven by both the strategy to build a sports powerhouse and the development of digital technology, China's sports broadcasting market has shown rapid growth. However, academic research has yet to systematically explore the underlying psychological mechanisms associated with the transition from “viewing” behavior to “participatory” consumption.

**Objective:**

Drawing on a management psychology perspective, this study aims to construct and test a sequential mediation model to examine the associations among Motivation for Watching Sports Events, emotional responses, psychological belonging, and consumption behavior.

**Methods:**

A questionnaire survey was conducted among a sample of 563 Chinese viewers with experience watching live sports broadcasting. Established scales were used to measure Motivation for Watching Sports Events, positive emotional responses, psychological belonging, and consumption behavior. Data analysis was performed using SPSS 26.0 (IBM Corp., Armonk, NY, USA) and AMOS 24.0 (IBM Corp., Armonk, NY, USA) software, and sequential mediation effects were examined using PROCESS macro Model 6 and the Bootstrap method.

**Results:**

The study found that: (1) motivation for Watching Sports Events was positively associated with consumption behavior (β = 0.575, p < 0.001) and showed indirect associations with consumption behavior through the mediating roles of emotional responses and psychological belonging, with mediation effect sizes of 0.114 and 0.088, respectively; (2) emotional responses and psychological belonging showed a significant sequential mediating effect in the association between viewing motivation and consumption behavior (effect size = 0.032). Specifically, viewing motivation was associated with positive emotional responses, which were further associated with psychological belonging and consumption behavior.

**Conclusion:**

This study supported the proposed “cognitive-emotional-identity-behavior” sequential mediation model and examined the psychological mechanisms underlying the association between “viewing” and “consumption.” The findings enrich the theoretical understanding of sports consumption behavior and provide practical insights for stakeholders in the sports industry regarding strategies to enhance viewers' emotional experiences and strengthen community belonging.

## Introduction

In recent years, the Chinese government has continuously increased its support for the sports industry, providing a favorable policy environment for the development of the sports consumption market. Policy documents successively issued by the State Council, including the “Several Opinions on Accelerating the Development of the Sports Industry and Promoting Sports Consumption” (State Council Document [2014] No. 46) and the “Outline for Building a Sports Powerhouse,” identify the sports industry as an important component of national economic development and establish the goal of developing it into a pillar industry by 2035 (Yu and Wang, 2021; Hu, 2015). Against this policy backdrop, the sports streaming market has experienced significant development opportunities. With the widespread application of technologies such as 5G, ultra-high-definition video, and virtual reality, sports streaming formats and audience viewing experiences have undergone substantial changes. According to statistics from the General Administration of Sport of China, the number of sports streaming users in China exceeded 500 million in 2022, indicating continued expansion of the sports streaming audience base (Jiang and Li, 2020). Sports streaming has not only changed viewers' media consumption patterns but has also been associated with emerging consumption patterns and business opportunities within the sports industry. Recent research has further highlighted the strategic role of social media in strengthening sponsorship effectiveness and audience engagement in sports events.

From an academic perspective, existing research has largely focused on single-dimensional analyses of either the communication effects or consumer behavior of sports live streaming, with limited exploration of the psychological processes associated with the transition from “viewing” behavior to Consumption Behavior. The mechanisms underlying sports live-streaming audiences' Consumption Behavior may involve complex psychological processes; examining these processes may further enrich theories in management psychology and inform sports marketing practices. Recent sponsorship research similarly suggests that audience engagement is associated with factors such as sport involvement, sponsor attitudes, and social media engagement, further highlighting the importance of psychological factors in sports-related consumer behavior. Against the backdrop of the rapid development of the digital economy, exploring the factors associated with sports streaming audiences' Consumption Behavior provides practical implications for optimizing business models and understanding sports consumption potential. Therefore, based on a management psychology perspective, this study constructs a theoretical model with Motivation for Watching Sports Events as the independent variable, Emotional Responses and Psychological Belonging as mediating variables, and Consumption Behavior as the dependent variable, to examine the psychological associations underlying sports streaming audiences' Consumption Behavior.

### The influence of motivation for watching sports events on consumption behavior

Motivation for Watching Sports Events represents an important psychological factor associated with viewers' viewing behavior and subsequent Consumption Behavior. According to Self-Determination Theory, an individual's motivation can be divided into two dimensions: intrinsic motivation and extrinsic motivation (Van den Broeck et al., 2021; Vallerand, 2000). Intrinsic motivation stems from the pleasure and satisfaction derived from the activity itself, such as enjoyment of and interest in sports competitions; extrinsic motivation, on the other hand, is related to external rewards that are separable from the activity, such as social needs or escapism (Legault, 2020; Morris et al., 2022). In the context of live sports broadcasting, viewers' motivations for watching are diverse, encompassing dimensions such as information acquisition, entertainment, social interaction, and self-improvement (Li, 2025; Majiri et al., 2025; Yang et al., 2025).

The relationship between Motivation for Watching Sports Events and Consumption Behavior may involve multiple psychological processes. According to the Stimulus-Organization-Response (S-O-R) theory (Duong, 2023; Sohaib et al., 2022), external stimuli (such as sports event content) are associated with individuals' internal states (cognitive and emotional), which are related to behavioral responses such as Consumption Behavior (Gârdan et al., 2020; Teal et al., 2020; Moital et al., 2019). Empirical studies indicate that the associations between different dimensions of Motivation for Watching Sports Events and Consumption Behavior vary. Related research has also shown that spectators and participants may differ in how they perceive sport sponsorship and form purchase intentions, suggesting that different forms of sport involvement may be associated with different consumer responses.

For example, viewers primarily motivated by information-seeking may show greater engagement in event-related information services or professional equipment purchases, whereas viewers primarily motivated by social interaction may demonstrate stronger participation in group-based consumption activities, such as organizing viewing parties or purchasing merchandise (Kim and Mao, 2021; Hua et al., 2023; Wang et al., 2024). Furthermore, the intensity of motivation is associated with differences in consumption frequency and amount. Viewers with high motivation intensity tend to report higher levels of brand loyalty and willingness to pay (Lopes and Galletta, 2006). Sport sponsorship research has similarly emphasized the importance of considering both consumers' attitudes and behavioral responses when evaluating sport-related consumption.

It is worth noting that the relationship between Motivation for Watching Sports Events and Consumption Behavior may not be explained solely by a direct association but may involve multiple psychological factors. Existing research has largely focused on the direct link between motivation and behavior, while the psychological processes involved in this relationship remain relatively underexplored. Therefore, this study incorporates Emotional Responses and Psychological Belonging into its analytical framework, aiming to examine whether these factors are associated with the relationship between Motivation for Watching Sports Events and Consumption Behavior.

### The relationship among motivation for watching sports events, emotional responses, and consumption behavior

Emotional responses—defined as an individual's subjective experience and physiological arousal in response to a stimulus—represent an important component of the consumer decision-making process. In the context of live sports broadcasting, the unpredictability of game progression and uncertainty of outcomes are often associated with intense emotional fluctuations among viewers (Breuer et al., 2021; Buraimo et al., 2020; Zhang et al., 2024). According to Appraisal Theory, an individual's cognitive evaluation of an event is related to the nature and intensity of emotional responses, which are subsequently associated with behavioral tendencies (Wondra and Ellsworth, 2015). Motivation for Watching Sports Events may be associated with differences in the intensity and direction of viewers' emotional responses by shaping their attention to and evaluation of in-game events.

Research indicates that emotional responses may serve as a mediating variable in the relationship between Motivation for Watching Sports Events and Consumption Behavior (Dang-Van et al., 2023). Viewers primarily motivated by identification tend to report stronger emotional investment in the outcomes of their favorite teams or athletes; emotions associated with victory or defeat are related to their consumption decisions (Yim and Byon, 2018). Positive emotions such as excitement and pride are associated with higher purchase intentions, whereas negative emotions such as disappointment and anger may be associated with compensatory consumption tendencies in certain circumstances (Miranda et al., 2025). For example, after their favorite team loses, fans may purchase team merchandise as a way of expressing support and identification.

The relationship between emotional responses and Consumption Behavior can also be understood from the perspectives of emotional contagion and emotional regulation. The collective viewing environment of live sports broadcasting—whether through in-person gatherings or online interactions—may facilitate emotional contagion; shared emotional experiences between individuals and groups are associated with consumption tendencies. At the same time, consumers may use Consumption Behavior to regulate or maintain specific emotional states, such as purchasing souvenirs to maintain positive feelings associated with the game experience (Gao et al., 2025). Thus, emotional responses may represent an important psychological pathway linking Motivation for Watching Sports Events and Consumption Behavior; examining this mediating relationship may provide further insight into viewers' consumption decisions.

### The relationship among motivation for watching sports events, psychological belonging, and consumption behavior

Psychological belonging refers to the emotional experience of being accepted, recognized, and included by a group, and it represents an important psychological need for individuals (Braun, 2019; Pardede and Kovač, 2023). In the context of live sports broadcasting, viewers may develop social identity and psychological belonging through identification with specific teams, athletes, or fan communities. According to Social Identity Theory, individuals incorporate group membership into their self-concept, which may be associated with in-group preference and perceptions of group boundaries (Banaji et al., 2021; Chater and Loewenstein, 2023; Tajfel et al., 2001). Different dimensions of Motivation for Watching Sports Events may be differently associated with psychological belonging. For example, viewers primarily motivated by social interaction may report stronger feelings of group belonging, whereas those primarily motivated by entertainment and recreation may show relatively weaker group identification.

The relationship between psychological belonging and Consumption Behavior can be discussed from three perspectives: group normative pressure, group identification, and group cohesion (Melnyk et al., 2022; Qian et al., 2021; Orazani et al., 2023; Sahharon et al., 2023). Group norms may be associated with individuals' adoption of Consumption Behavior consistent with the group, while group identification and cohesion may correspond with greater engagement in group-related products, services, and collective consumption activities. Empirical studies indicate that sports fans with a strong sense of belonging tend to report greater willingness to purchase authentic jerseys, participate in game-day events, and pay a premium for paid content subscriptions (Hedlund, 2017). Consumption Behavior associated with psychological belonging may satisfy functional needs while also reflecting aspects of social identity.

The relationship between psychological belonging and Consumption Behavior may vary across cultural contexts. Chinese audiences in collectivist cultural contexts may place greater emphasis on group belonging compared with audiences in more individualistic cultural contexts (Yu et al., 2025). Furthermore, virtual communities and fan groups in the new media environment provide additional contexts in which psychological belonging may develop. The immediacy and breadth of online interactions are associated with viewers' sense of participation and belonging, which may also be related to Consumption Behavior. Therefore, examining the mediating role of psychological belonging between Motivation for Watching Sports Events and Consumption Behavior may contribute to understanding the psychological factors associated with sports streaming consumption in the Chinese context.

### The chain mediation of emotional responses and psychological belonging

Emotional responses and psychological belonging are related psychological factors that may jointly explain variations in sports streaming audiences' Consumption Behavior. According to Cognitive-Affective System Theory, an individual's cognition, emotions, and motivation constitute a system in which these psychological components are interconnected (Mischel and Shoda, 1995; Thielmann et al., 2020; Tett et al., 2021). In the context of live sports broadcasting, viewers' emotional responses may be associated with differences in psychological belonging, which may also be related to consumption decisions. For example, shared emotional experiences, such as celebrating a team's victory together, may be associated with stronger group cohesion and a greater sense of psychological belonging (Diederik Coetzee and Hoch, 2024).

Emotional responses and psychological belonging may form a sequential mediation model, in which the associations among Motivation for Watching Sports Events, emotional responses, psychological belonging, and Consumption Behavior are examined sequentially. This sequential mediation model represents a theoretical explanation of the relationship between emotional arousal, social identification, and Consumption Behavior. Empirical research indicates that intense emotional experiences, especially positive emotions, are associated with stronger group identification and belonging, which are also associated with group-related Consumption Behavior (Mutz and Gerke, 2018). For example, the excitement and pride fans experience after witnessing their team's come-from-behind victory may correspond with stronger fan identity and greater willingness to purchase team merchandise.

Moreover, the connections between emotional responses and psychological belonging might differ according to each situation. For example, during stressful events like critical games or home games, the emotional ups and downs can be more pronounced, and the link between emotional responses and psychological belonging can be stronger. new media platforms have interactive capabilities, such as real-time commenting and virtual gifts, which could offer new possibilities for emotional expression and be linked to a stronger sense of psychological belonging. Thus, it is worth considering the sequential mediating role of emotional responses and psychological belonging to better understand the psychological variables related to sports streaming audiences' Consumption Behavior and the connection between sport viewing and Consumption Behavior.

## Research hypotheses

Based on the theoretical analysis and literature review presented above, this study proposes the following research hypotheses (see Figure 1):

**Figure 1:**
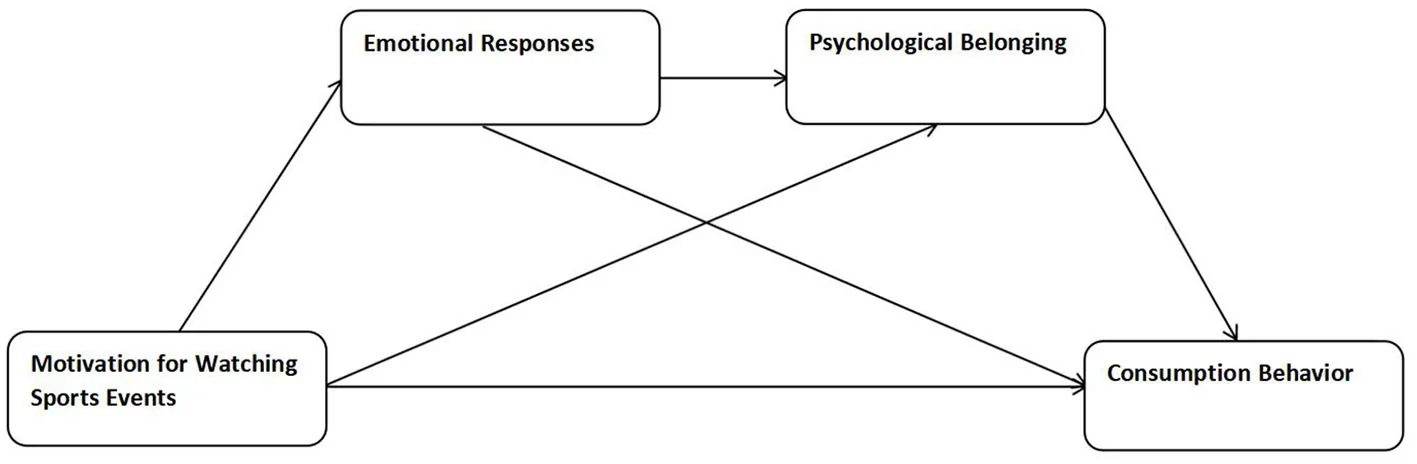
Schematic diagram of the hypothesis model.

- **H1:** Motivation for watching sports events is positively associated with consumption behavior.
- **H2:** Emotional responses mediate the association between motivation for watching sports events and consumption behavior.
- **H3:** Psychological belonging mediates the association between motivation for watching sports events and consumption behavior.
- **H4:** Emotional responses and psychological belonging sequentially mediate the association between motivation for watching sports events and consumption behavior.

By testing these hypotheses, this study aims to examine a model of sports streaming audiences' Consumption Behavior, explore the psychological factors associated with the relationship between “viewing” and “consumption,” and provide empirical evidence for sports consumption research from a management psychology perspective.

## Research methodology

### Research participants

The present study is a questionnaire survey which aims to investigate the relationship between sport live-streaming viewing and consumption behavior of the audiences. the subjects were chinese fans who have watched live sports events, such as soccer, basketball, and esports, in the last 6 months.

Online convenience sampling and snowball sampling were used in the sampling process. The questionnaire was specifically developed on the Internet survey platform Wenjuanxing and distributed via the following channels: (1) posting recruitment notices in sports related groups or communities on social media such as Weibo and Douban; (2) inviting college sports groups to distribute the questionnaire to their members and (3) distributing the questionnaire through the personal social media of the members of the research team, such as WeChat Moments and QQ groups.

Data collection was conducted from December 2025 to March 2026. To ensure sample relevance, a screening question (“Have you watched a live sports event in the past 6 months?”) was included at the beginning of the questionnaire, and respondents who answered “No” were excluded. A total of 612 questionnaires were collected. After removing invalid responses, including questionnaires completed in < 150 s, those showing repetitive response patterns (e.g., selecting the same option for all items), and those failing the attention check, 563 valid questionnaires remained, yielding a valid response rate of 92.0%.

Among the valid respondents, males accounted for 50.3% (*N* = 317), and females accounted for 49.7% (*N* = 246). The sample mainly consisted of young and middle-aged adults, with participants aged 18–25 accounting for 37.3%, those aged 26–35 accounting for 29.0%, and those aged 36 and above accounting for 33.7%. Regarding educational background, respondents with a bachelor's degree or above represented the largest proportion (62.7%). In terms of viewing frequency, 65.4% of participants watched live sports broadcasts at least once per week, suggesting that the sample primarily consisted of relatively active sports viewers rather than representing the full spectrum of sports streaming audiences in China. This characteristic should be taken into consideration when interpreting the findings and their generalizability. The research protocol was approved by the ethics committee of the authors' university, and all participants provided informed consent before participation (No. 2025-12).

### Research instruments

This study used established scales for data collection. All items were measured using a 5-point Likert scale ranging from 1 (“strongly disagree”) to 5 (“strongly agree”). Before the formal survey, two psychology experts and one sports science expert conducted translation and back-translation of the scale items to ensure their suitability for the Chinese context and content validity.

### Sports spectator motivation scale

The Chinese version of the Sports Spectator Motivation Scale developed by Trail and James (2001) and revised by Xie et al. (2018) was adopted. The scale contains 24 items covering six dimensions: achievement motivation, knowledge motivation, aesthetic motivation, social motivation, family motivation, and escape motivation. Example items include “I watch sports to experience the thrill of competition” (achievement motivation) and “I watch sports to learn new athletic skills” (knowledge motivation).

In this study, the overall Cronbach's α coefficient was 0.942, indicating good internal consistency reliability. Confirmatory factor analysis showed satisfactory construct validity, with χ^2^/df = 2.841, CFI = 0.953, TLI = 0.941, RMSEA = 0.058, and SRMR = 0.041. Standardized factor loadings ranged from 0.632 to 0.856, and the composite reliability was 0.937.

### Emotional responses scale

The Chinese revised version of the Positive and Negative Affect Schedule (PANAS) developed by Watson et al. (1988) and adapted by (Chen and Zhang 2004) was adopted, with item wording adjusted to fit the context of live sports broadcasting. The scale consists of 10 items measuring positive emotions (five items, such as “excited” and “enthusiastic”) and negative emotions (five items, such as “depressed” and “annoyed”).

This study focuses on the mediating role of positive emotional responses in the association between Motivation for Watching Sports Events and Consumption Behavior. The Cronbach's α coefficient of the positive emotion subscale was 0.916. Confirmatory factor analysis indicated a satisfactory unidimensional structure, with χ^2^/df = 2.105, CFI = 0.987, TLI = 0.978, RMSEA = 0.044, and SRMR = 0.027. Standardized factor loadings exceeded 0.70, and the composite reliability was 0.918.

### Psychological belonging scale

The Sports Spectator Psychological Belonging Scale, developed by Malone et al. (2012) based on social identity theory and validated in the Chinese context by Zhu and Feng (2024), was adopted. The scale consists of six items measuring spectators' identification with and belonging to fan communities. Example items include “I feel like I am part of this fan community” and “I have a strong sense of belonging to this fan community.”

The Cronbach's α coefficient of this scale was 0.889. Confirmatory factor analysis showed satisfactory model fit, with χ^2^/df = 2.567, CFI = 0.981, TLI = 0.968, RMSEA = 0.053, and SRMR = 0.032. Standardized factor loadings ranged from 0.681 to 0.802, and the composite reliability was 0.892.

### Consumption behavior scale

The Consumption Behavior Scale was developed based on previous studies by Funk and James (2001) and Yoshida et al. (2018), while considering characteristics of the Chinese sports consumption market, such as tipping during live streams and paid memberships. The scale consists of five items measuring viewers' consumption behavior related to live sports broadcasting. The items cover purchasing merchandise, purchasing paid viewing packages, attending live events, tipping or sending gifts on live-streaming platforms, and participating in offline sports-related consumption activities associated with sporting events. Although these activities differ in their specific forms, they all represent behavioral manifestations of sports-related consumption arising from sports spectatorship. Specifically, they reflect viewers' overall propensity to convert sports viewing experiences into economic participation through multiple consumption channels. Therefore, consistent with previous research on sport consumer behavior, these activities were conceptualized as indicators of a single latent construct representing overall sports Consumption Behavior rather than as independent consumption outcomes.

The Cronbach's α coefficient of this scale was 0.865. Confirmatory factor analysis indicated a unidimensional structure, with χ^2^/df = 2.893, CFI = 0.972, TLI = 0.955, RMSEA = 0.058, and SRMR = 0.035. Standardized factor loadings exceeded 0.60, and the composite reliability was 0.869. These results provide empirical support for the unidimensionality of the scale, indicating that the five consumption activities can be adequately represented by a single latent construct of sports Consumption Behavior.

## Results

### Common method bias test

Because all four key variables were collected from the same respondents using self-report questionnaires administered at the same time point, common method variance (CMV) was identified as a potential source of systematic bias. To address this concern, two complementary procedures were employed.

### Procedural control

First, several procedural remedies were implemented during data collection to reduce the likelihood of common method variance, as recommended by Podsakoff et al. (2003). The questionnaire used different scale anchors (e.g., “strongly disagree–strongly agree” for some constructs, “never–always” for others) and separated predictor and criterion variables across non-adjacent sections of the questionnaire. Respondents were guaranteed anonymity, and no personally identifiable information was collected.

### Harman's single-factor test

Second, Harman's single-factor test was conducted by loading all items of the four study variables into an unrotated exploratory factor analysis in SPSS 26.0. The results showed that the first unrotated factor accounted for 38.42% of the total variance, which is well below the 50% threshold recommended in the literature (Podsakoff et al., 2003; Podsakoff and Organ, 1986). The first five factors together explained 67.85% of the variance, with eigenvalues of 11.52, 4.28, 3.16, 2.71, and 1.86, respectively. These results indicate that no single factor accounted for the majority of the covariance among the items, suggesting that common method bias was not a serious threat to the present study.

### Common latent factor test

As a more rigorous supplementary check, a common latent factor (CLF) model was specified in AMOS 24.0. All items loaded on both their substantive latent factor and a common latent factor. The standardized regression weights from the substantive factors were compared with and without the CLF. The differences were small (Δ < 0.10), indicating that common method variance did not substantially inflate the observed relationships.

### Measurement model and discriminant validity

To address the concern regarding the high intercorrelations among the four main constructs, a comprehensive set of measurement-model diagnostics was conducted in addition to the per-scale CFA reported above. The full four-factor measurement model, with all observed items loading on their corresponding latent factors (Motivation for Watching Sports Events, Emotional Responses, Psychological Belonging, and Consumption Behavior), was estimated using AMOS 24.0 with maximum likelihood estimation.

The complete measurement model yielded acceptable fit to the data: χ^2^/df = 2.412, CFI = 0.946, TLI = 0.941, RMSEA = 0.051 (90% CI [0.048, 0.054]), and SRMR = 0.045. All standardized factor loadings were significant (*p* < 0.001) and ranged from 0.612 to 0.871. Table 1 reports the convergent and discriminant validity evidence.

**Table 1:** Convergent and discriminant validity of the measurement model.

Construct	CR	AVE	√AVE	1. MWSE	2. ER	3. PB	4. CB
1. MWSE	0.937	0.612	0.782	—	0.793^**^	0.798^**^	0.807^**^
2. ER	0.918	0.575	0.758	0.793^**^	—	0.733^**^	0.711^**^
3. PB	0.892	0.682	0.826	0.798^**^	0.733^**^	—	0.714^**^
4. CB	0.869	0.624	0.790	0.807^**^	0.711^**^	0.714^**^	—

MWSE, motivation for watching sports events; ER, emotional responses; PB, psychological belonging; CB, consumption behavior. The double asterisks (**) indicate statistical significance at the 0.01 level (*p* < 0.01).

### Convergent validity

Convergent validity was assessed via standardized factor loadings, CR, and AVE (Hair et al., 2019). As shown in Table 1, all CR values exceeded the recommended threshold of 0.70 (range = 0.869–0.937) and all AVE values exceeded the recommended threshold of 0.50 (range = 0.575–0.682), supporting convergent validity.

### Discriminant validity—Fornell–Larcker criterion

According to the Fornell–Larcker criterion (Fornell and Larcker, 1981), discriminant validity is supported when the square root of the AVE for each construct exceeds its correlations with the other constructs. As shown in Table 1, the diagonal √AVE values (0.758–0.826) were all larger than the corresponding off-diagonal inter-construct correlations (0.711–0.807), confirming discriminant validity under the Fornell–Larcker criterion.

### Discriminant validity—HTMT

Because the Fornell–Larcker criterion has been criticized as being insensitive in detecting a lack of discriminant validity, the heterotrait-monotrait (HTMT) ratio of correlations was also computed following (Henseler et al., 2015). Table 2 reports the HTMT values for all pairs of constructs.

**Table 2:** HTMT ratios for discriminant validity.

Construct pair	HTMT	Threshold	Conclusion
MWSE ↔ ER	0.812	< 0.85	Supported
MWSE ↔ PB	0.821	< 0.85	Supported
MWSE ↔ CB	0.842	< 0.85	Supported
ER ↔ PB	0.759	< 0.85	Supported
ER ↔ CB	0.748	< 0.85	Supported
PB ↔ CB	0.762	< 0.85	Supported

### Multicollinearity diagnostics

Although the correlations among the four constructs are moderate-to-high, variance inflation factor (VIF) and tolerance diagnostics indicated that multicollinearity was not at a problematic level for the structural model. Table 3 reports the VIF and tolerance values for each predictor in the regression equations.

**Table 3:** Multicollinearity diagnostics (VIF and tolerance).

Dependent variable	Predictor	Tolerance	VIF
Emotional responses (M1)	Motivation for watching sports events	0.412	2.43
Psychological belonging (M2)	Motivation for watching sports events	0.362	2.76
Psychological belonging (M2)	Emotional responses	0.418	2.39
Consumption behavior (Y)	Motivation for watching sports events	0.339	2.95
Consumption behavior (Y)	Emotional responses	0.428	2.34
Consumption behavior (Y)	Psychological belonging	0.385	2.60

All VIF values were below the conservative threshold of 5.0 (Hair et al., 2019) and tolerance values exceeded 0.20, indicating that multicollinearity did not inflate the standard errors or distort the parameter estimates in the structural model.

### Correlation analysis

Descriptive statistics and Pearson correlation analyses were conducted for all study variables, and the results are presented in Table 4. Descriptive statistics indicated that the mean values of the four variables ranged from 2.394 to 2.472, with standard deviations ranging from 0.834 to 0.932. Pearson correlation analysis showed significant positive correlations among Motivation for Watching Sports Events, Emotional Responses, Psychological Belonging, and Consumption Behavior (*r* = 0.711–0.807, *p* < 0.01).

**Table 4:** Descriptive statistics and correlation matrix of study variables.

Variable	*M*	SD	1	2	3	4
1. Motivation for watching sports events	2.408	0.834	1			
2. Emotional responses	2.413	0.917	0.793^**^	1		
3. Psychological belonging	2.472	0.889	0.798^**^	0.733^**^	1	
4. Consumption behavior	2.394	0.932	0.807^**^	0.711^**^	0.714^**^	1

The double asterisks (**) indicate statistical significance at the 0.01 level (*p* < 0.01).

### Testing the sequential mediation model

Following Hayes's (2018) mediation analysis procedure, the PROCESS macro (Model 6) with bootstrap estimation was used to examine the sequential mediation model involving Emotional Responses and Psychological Belonging. Gender, age, and educational attainment were included as control variables. The results are presented in Table 5 and Figure 2.

**Table 5:** Regression results of the sequential mediation model.

Dependent variable	Predictor	β	*t*	*p*
Emotional responses (M1)	Motivation for watching sports events (X)	0.796	30.999	< 0.001
	Gender	0.003	0.119	0.905
	Age	−0.010	−0.372	0.710
	Educational attainment	−0.072	−2.813	0.005
Psychological belonging (M2)	Motivation for watching sports events (X)	0.585	14.432	< 0.001
	Emotional responses (M1)	0.269	6.640	< 0.001
	Gender	0.020	0.805	0.421
	Age	0.006	0.243	0.808
	Educational attainment	−0.006	−0.249	0.804
Consumption behavior (Y)	Motivation for watching sports events (X)	0.575	12.280	< 0.001
	Emotional responses (M1)	0.143	3.439	0.001
	Psychological belonging (M2)	0.151	3.605	< 0.001
	Gender	0.007	0.278	0.781
	Age	−0.025	−1.035	0.301
	Educational attainment	−0.042	−1.741	0.082
Total effect model	Motivation for watching sports events (X)	0.809	32.484	< 0.001
	Gender	0.010	0.412	0.680
	Age	−0.026	−1.041	0.298
	Educational attainment	−0.057	−2.274	0.023

β values represent standardized regression coefficients. All variables included in the model were standardized before analysis.

**Figure 2:**
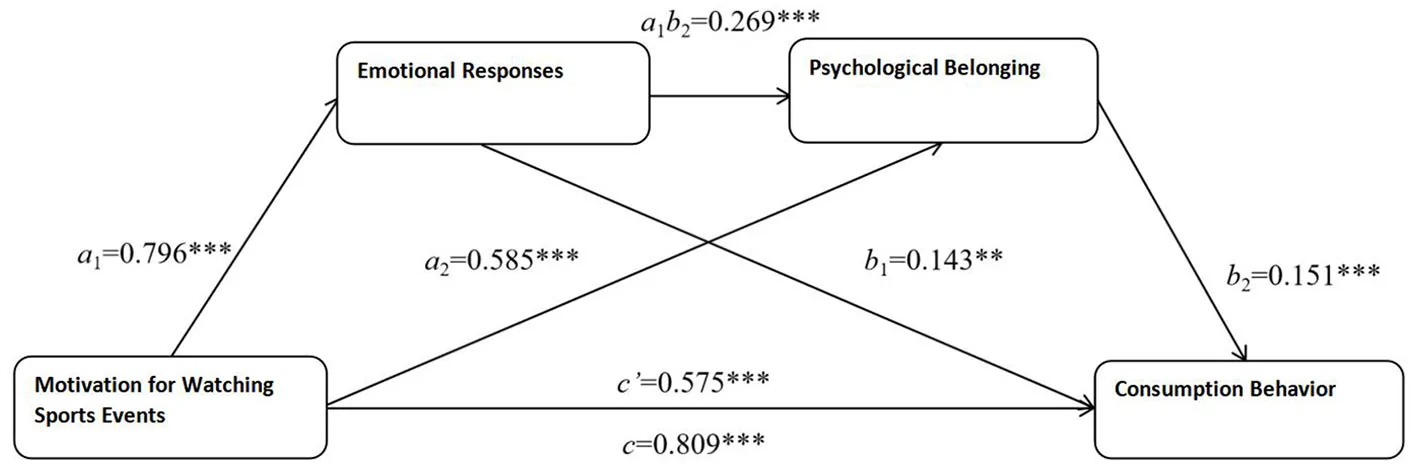
Model verification diagram. The values represent standardized path coefficients. ** indicates *p* < 0.01, and *** indicates *p* < 0.001.

The standardized regression coefficients showed that Motivation for Watching Sports Events was positively associated with Emotional Responses (β = 0.796, *t* = 30.999, *p* < 0.001). When Psychological Belonging was examined as the dependent variable, both Motivation for Watching Sports Events (β = 0.585, *t* = 14.432, *p* < 0.001) and Emotional Responses (β = 0.269, *t* = 6.640, *p* < 0.001) showed significant positive associations with Psychological Belonging.

In the final model predicting Consumption Behavior, Motivation for Watching Sports Events (β = 0.575, *p* < 0.001), Emotional Responses (β = 0.143, *p* = 0.001), and Psychological Belonging (β = 0.151, *p* < 0.001) remained significantly associated with Consumption Behavior. The total association between Motivation for Watching Sports Events and Consumption Behavior was also significant (β = 0.809, *p* < 0.001).

The coefficients of the control variables were generally smaller than those of the main variables, and most were not statistically significant.

### Bootstrap test of indirect effects and hypothesis summary

The indirect effects were examined using the bootstrap method with 5,000 resamples. The total indirect effect of Motivation for Watching Sports Events on Consumption Behavior was 0.234, with a 95% bootstrap confidence interval of [0.154, 0.316]. Because the confidence interval did not include zero, the overall indirect effect was statistically significant (see Table 6).

**Table 6:** Bootstrap test results for direct, indirect, and total effects.

Pathway	Effect	Boot SE	95% Boot CI lower	95% Boot CI upper
Total effect (MWSE → CB)	0.809	0.025	0.760	0.858
Total indirect effect	0.234	0.042	0.154	0.316
Indirect 1: MWSE → ER → CB	0.114	0.039	0.039	0.190
Indirect 2: MWSE → PB → CB	0.088	0.028	0.034	0.145
Indirect 3: MWSE → ER → PB → CB	0.032	0.013	0.010	0.061
Direct effect (MWSE → CB)	0.575	0.047	0.483	0.667

Bootstrap sample = 5,000.

Further examination of the specific indirect effects showed that all three indirect pathways were significant (See Table 7): (1) the indirect effect through Emotional Responses was 0.114, 95% bootstrap CI [0.039, 0.190]; (2) the indirect effect through Psychological Belonging was 0.088, 95% bootstrap CI [0.034, 0.145]; and (3) the sequential indirect effect through Emotional Responses and Psychological Belonging was 0.032, 95% bootstrap CI [0.010, 0.061]. After including the mediators, the direct effect of Motivation for Watching Sports Events on Consumption Behavior remained significant (effect = 0.575, 95% bootstrap CI [0.483, 0.667]).

**Table 7:** Summary of hypothesis testing.

Hypothesis	Pathway/Effect	β/Effect	95% CI	Supported
H1	Direct effect: MWSE → CB	0.575	[0.483, 0.667]	Yes
H2	Indirect 1: MWSE → ER → CB	0.114	[0.039, 0.190]	Yes
H3	Indirect 2: MWSE → PB → CB	0.088	[0.034, 0.145]	Yes
H4	Indirect 3: MWSE → ER → PB → CB	0.032	[0.010, 0.061]	Yes
—	Total indirect effect	0.234	[0.154, 0.316]	—
—	Total effect (MWSE → CB)	0.809	[0.760, 0.858]	—

H1 was evaluated by the direct path coefficient (β); H2–H4 were evaluated by the 95% bootstrap confidence interval excluding zero. The direct effect (β = 0.575) remained significant after introducing the mediators, indicating partial mediation.

## Discussion

From a management psychology perspective, this study developed a theoretical model with Motivation for Watching Sports Events as the independent variable, Emotional Responses and Psychological Belonging as mediating variables, and Consumption Behavior as the dependent variable. Based on questionnaire data from 563 Chinese live sports viewers, the findings generally support the proposed theoretical framework and suggest that viewers' motivation is associated with sports consumption both directly and indirectly through emotional and social psychological processes. Rather than operating independently, Emotional Responses and Psychological Belonging appear to represent complementary psychological mechanisms through which viewing experiences are translated into consumption participation. This finding provides a more integrated understanding of the psychological processes linking sports viewing and Consumption Behavior in the context of digital sports broadcasting.

### The relationship between motivation for watching sports events and consumption behavior

The positive association between Motivation for Watching Sports Events and Consumption Behavior can be understood from the perspective of Self-Determination Theory. This theory distinguishes intrinsic motivation, which originates from enjoyment and personal satisfaction, from extrinsic motivation, which is driven by external rewards and social goals (Van den Broeck et al., 2021; Vallerand, 2000). In sports live-streaming contexts, these motivational dimensions often coexist. Viewers may initially be attracted by the excitement of competition or the pursuit of sports knowledge, but continued engagement may gradually strengthen their willingness to invest time, money, and other resources in sports-related activities. Consequently, Consumption Behavior can be viewed as an extension of sustained motivational engagement rather than merely an isolated purchasing decision. In the context of live sports broadcasting, viewers' motivations include multiple dimensions, such as information acquisition, entertainment, and social interaction (Li, 2025; Majiri et al., 2025; Yang et al., 2025).

The findings of this study are consistent with previous research. For example, Kim and Mao (2021) reported that viewers motivated by information acquisition showed greater engagement with professional equipment purchases, whereas viewers motivated by social interaction were more involved in group-based consumption activities. In addition, the Stimulus-Organism-Response (S-O-R) theory suggests that external stimuli are associated with individuals' internal cognitive and emotional states, which are related to behavioral responses (Duong, 2023; Sohaib et al., 2022). From this perspective, viewing motivation may correspond with differences in viewers' cognitive evaluation and emotional engagement, which are associated with consumption decisions (Gârdan et al., 2020; Moital et al., 2019). Consistent with previous findings, viewers with stronger motivation tend to report higher brand loyalty and willingness to pay (Lopes and Galletta, 2006).

From a management psychology perspective, this finding highlights the importance of motivational processes in understanding sports consumption. Compared with previous studies that mainly focused on the direct relationship between viewing motivation and consumer behavior, the present study further demonstrates that the influence of viewing motivation should be understood within a broader psychological framework involving emotional and social mechanisms.

### The mediating role of emotional responses

The mediating role of Emotional Responses between Motivation for Watching Sports Events and Consumption Behavior can be explained by Appraisal Theory. This theory suggests that individuals' evaluations of events are related to the nature and intensity of their emotional responses, which may subsequently be associated with behavioral outcomes (Wondra and Ellsworth, 2015). In the context of live sports broadcasting, the uncertainty of game outcomes and the changing dynamics of competition are often associated with strong emotional experiences among viewers (Breuer et al., 2021; Buraimo et al., 2020; Zhang et al., 2024).

The findings of this study indicate that Motivation for Watching Sports Events is indirectly associated with Consumption Behavior through Emotional Responses This result is consistent with Dang-Van et al. (2023), who suggested that emotional responses may serve as an important psychological factor connecting motivation and behavioral outcomes. Similarly, Yim and Byon (2018) found that emotional experiences among sports fans were related to stronger consumption intentions following team victories.

Emotional contagion and emotional regulation provide additional explanations for the relationship between Emotional Responses and Consumption Behavior. In live sports broadcasting environments, whether through offline gatherings or online interactions, shared emotional experiences among viewers may strengthen emotional connections and increase engagement with related consumption activities. Meanwhile, consumers may engage in Consumption Behavior as a way of maintaining emotional connections with sporting events, teams, or athletes, such as purchasing souvenirs to preserve positive experiences associated with a game (Gao et al., 2025).

In this study, the indirect effect of Emotional Responses was significant, indicating that emotional experiences represent an important psychological factor associated with the relationship between viewing motivation and Consumption Behavior. These findings suggest that sports organizations and platforms may consider emotional engagement as an important element when designing viewer experiences and consumption environments.

### The mediating role of psychological belonging

The mediating role of Psychological Belonging between Motivation for Watching Sports Events and Consumption Behavior can be understood from the perspective of Social Identity Theory. This theory suggests that individuals incorporate group membership into their self-concept, which is associated with in-group identification and feelings of belonging (Tajfel et al., 2001; Banaji et al., 2021). In the context of live sports broadcasting, viewers may develop Psychological Belonging through identification with specific teams, athletes, or fan communities, and this sense of belonging may be related to their consumption decisions.

The results of this study showed that the indirect effect of Psychological Belonging was significant. This finding suggests that Psychological Belonging partially explains the association between Motivation for Watching Sports Events and Consumption Behavior. The result is consistent with Hedlund (2017), who found that sports viewers with stronger feelings of belonging reported greater willingness to purchase authentic jerseys and subscribe to paid content.

There are three aspects that can explain the relationship between Psychological Belonging and Consumption Behavior. First, group norms can affect the preferences of individuals as regards their Consumption Behavior, which is compatible with their fan communities (Melnyk et al., 2022). Second, people are more willing to pay for products and services that they connect with their group membership (Qian et al., 2021). Third, membership in a group can be linked to an increase in the use of collective consumption activities (Orazani et al., 2023; Sahharon et al., 2023).

The relationship between Psychological Belonging and Consumption Behavior might also be influenced by cultural context. In collectivist cultures, like China, people might prioritize a sense of belonging and group identity in consumption practices (Yu et al., 2025). In addition, virtual communities in the new media environment provide additional contexts for developing Psychological Belonging. The instantness and accessibility of the online interaction can enhance viewers' participation experiences and be linked to belonging feelings (Sahharon et al., 2023).

The mediating role of Psychological Belonging was also found to be significant and therefore social identification is an important psychological variable in the context of sports Consumption Behavior in China.

### The sequential mediation of emotional responses and psychological belonging

The sequential mediation of Emotional Responses and Psychological Belonging can be interpreted from the perspective of Cognitive-Affective System Theory. This theory suggests that individuals' cognition, emotions, and motivation constitute an interconnected system involving multiple psychological components (Mischel and Shoda, 1995; Thielmann et al., 2020). The findings support the proposed sequential mediation model and suggest that Emotional Responses and Psychological Belonging jointly explain the association between viewing motivation and Consumption Behavior. This indicates that viewers' emotional experiences may strengthen their sense of belonging, which is further associated with sports-related consumption.

The sequential mediation model provides a theoretical explanation of the relationship between emotional experiences, social identification, and consumption behavior. Previous research has shown that intense emotional experiences, such as shared excitement following a team victory, are associated with stronger group identity and feelings of belonging, which are also related to group-related consumption behavior (Mutz and Gerke, 2018). In live sports broadcasting environments, emotional contagion may represent an important factor connecting individual experiences with group identification. Shared emotional experiences among viewers, such as celebrating a victory together, may correspond with stronger group cohesion and Psychological Belonging (Diederik Coetzee and Hoch, 2024).

The relationship between Emotional Responses and Psychological Belonging may vary across different viewing contexts. High-stakes competitions, such as important matches or local derbies, may generate stronger emotional experiences, and these experiences may be more closely associated with feelings of belonging (Zhang et al., 2024). Interactive features on new media platforms, such as virtual gifts and real-time comments, may provide additional opportunities for emotional expression and social interaction.

Overall, the findings highlight the importance of considering emotional experiences and social identification as interconnected psychological processes rather than independent factors. Future research could further examine how contextual factors and digital interaction environments shape these relationships. Given the cross-sectional nature of the present study, the proposed sequential relationship should be interpreted as a theoretically supported association rather than a confirmed causal process.

### Research limitations and future directions

This study offers a number of insights, but also some limitations should be noted. One is that the findings may not be generalizable as the sampling was done online and via snowball sampling. The majority of the sample was young and middle-aged adults, which might not be representative of all live-streaming sport fans. As a result, the findings should be interpreted as reflecting the characteristics of the surveyed participants rather than the broader population of sports streaming audiences. Future studies could use a probability sampling technique to increase sample representativeness.

Secondly, the cross-sectional design makes it difficult to draw causal conclusions. Longitudinal or experimental designs might be more suitable to strengthen the evidence of the temporal relationship between viewing motivation, Emotional Responses, Psychological Belonging, and Consumption Behavior, as this is in line with the proposed theoretical model, which is sequential. Therefore, the proposed sequential mediation model should be interpreted as reflecting statistically supported associations rather than definitive causal relationships.

Third, all measures were self-report scales, which could be subject to possible response bias. Although common method bias was assessed and the results suggested that it was not a serious concern, the use of self-reported data may still have influenced participants' responses because of social desirability or subjective perceptions. In future research, subjective measures could be used alongside objective measures of behavior, e.g., actual consumption records, or physiological measures associated with emotional response.

Fourth, this study only identified Emotional Responses and did not distinguish between positive and negative emotions. Previous studies indicate that negative emotions like disappointment or anger are also potentially related to sport consumption behavior (Miranda et al., 2025). Consequently, the proposed model may not fully capture the complexity of emotional mechanisms underlying sports consumption. Future research could involve the study of various emotional aspects together.

Fifth, the findings should also be interpreted within the Chinese cultural context. Cultural context needs to be explored further. Research comparing different cultural groups' consumption of sport might yield further insights into the connections between social identity, belonging, and sport consumption. Cross-cultural comparisons would also help determine whether the proposed psychological mechanisms are applicable across different cultural settings.

Moreover, this research did not differentiate between sports such as esports, soccer, and basketball. Different sports may evoke different levels of emotional engagement, fan identification, and consumption patterns. Therefore, caution should be exercised when extending the present findings to specific sports contexts. Future research could examine whether different sports are associated with different psychological mechanisms. Lastly, other variables may be added in the future, such as individual differences (e.g., personality traits) and environmental factors (e.g., social media interaction), to provide a more comprehensive understanding of sports consumption behavior.

### Theoretical and practical implications

Theoretical and practical implications of this study have been mentioned. Theoretically, it combines Self-Determination Theory, Stimulus-Organism-Response (S-O-R) Theory, and Social Identity Theory to analyze psychological factors of sports streaming consumption behavior. This study contributes to the current literature on Motivation for Watching Sports Events and Consumption Behavior by adding two mediating variables: Emotional Responses and Psychological Belonging.

The fact that a sequential mediation effect is identified adds further weight to the importance of emotional experiences and social identification in comprehending sports consumption. The results also support the use of CAS in understanding the consumption of sport, as more than one psychological factor is found to be relevant in the understanding of Consumption Behavior.

## Conclusion

This study examined the relationship between Motivation for Watching Sports Events and Consumption Behavior among sports streaming audiences from a management psychology perspective. The findings indicate that viewing motivation is positively associated with consumption behavior and that this relationship can be partly explained by Emotional Responses and Psychological Belonging.

Specifically, the results show that Emotional Responses and Psychological Belonging individually and sequentially mediate the association between viewing motivation and consumption behavior. The sequential mediation model suggests that emotional experiences and social identification represent important psychological factors related to sports consumption.

Overall, this study contributes to understanding how psychological factors are associated with the relationship between sports viewing and consumption. By integrating motivational, emotional, and social identity perspectives, the findings provide theoretical and practical insights for future research and management practices in the sports consumption context.

## Data Availability

The raw data supporting the conclusions of this article will be made available by the authors, without undue reservation.
